# Bioinspired Soot‐Deposited Janus Fabrics for Sustainable Solar Steam Generation with Salt‐Rejection

**DOI:** 10.1002/gch2.201800117

**Published:** 2019-02-10

**Authors:** Shouwei Gao, Xiuli Dong, Jianying Huang, Jianing Dong, Francesco Di Maggio, Shanchi Wang, Fang Guo, Tianxue Zhu, Zhong Chen, Yuekun Lai

**Affiliations:** ^1^ National Engineering Laboratory for Modern Silk College of Textile and Clothing Engineering Soochow University Suzhou 215123 P. R. China; ^2^ College of Chemical Engineering Fuzhou University Fuzhou 350116 P. R. China; ^3^ Division of Medicine University College London London WC1E 6JF UK; ^4^ School of Materials Science and Engineering Nanyang Technological University 50 Nanyang Avenue Singapore 639798 Singapore

**Keywords:** Janus, salt‐rejection, soot, superhydrophobicity, water vapor generation

## Abstract

Inspired by lotus leaves, self‐floating Janus cotton fabric is successfully fabricated for solar steam generation with salt‐rejecting property. The layer‐selective soot‐deposited fabrics not only act as a solar absorber but also provide the required superhydrophobicity for floating on the water. With a polyester protector, the prepared Janus evaporator exhibits a sustainable evaporation rate of 1.375 kW m^−2^ h^−1^ and an efficiency of 86.3% under 1 sun (1 kW m^−2^) and also performs well under low intensity and inclined radiation. Furthermore, no special apparatus and/or tedious processes are needed for preparing this device. With a cost of less than $1 per m^2^, this flexible Janus absorber is a promising tool for portable solar vapor generator.

Water scarcity has become a global issue due to the fast‐growing population and rapid increase of pollution from industrial activities. More than 1.2 billion people lack access to safe drinking water around the world.^1^, ^2^ Two most common solutions are implemented to solve this problem. The first one involves the purification of wastewater, while the second one depends on seawater desalination. However, the use of commercial desalination method as well as the wastewater purification is limited due to its long processes, expensive equipment and high energy consumption.^3^, ^4^, ^5^ So it is urgent to search for new methods to solve this problem. A possible solution requires the use of abundant solar energy to produce heat for vapor generation.^6^, ^7^, ^8^, ^9^, ^10^, ^11^, ^12^ In that case, the water can get energy from sun to vaporize and thus realize the purification of wastewater or sea in an easy and cheap way. Among this, the interfacial heat localization takes advantage of a floating structure to focus the heat on the surface, which can avoid heating the bulk water and decrease the loss of heat, thus realizing high‐efficiency solar evaporation.

To focus the heat on the surface, the absorber should float on the surface of the water, so the first thing is to prepare the absorber with a self‐floating property. In this regards, lotus leaf provides a good example. In the nature, the lotus leaves can float on the air–water interface steadily even in a stormy weather. Cao and co‐workers found that the downside of the leaf is superhydrophilic while for the upside, due to the epicuticular wax crystalloids, it exhibits a great superhydrophobic property. Such special Janus wetting nature endows the lotus with such a prominent stability.^13^ The second is to choose cheap but excellent light absorb materials which can absorb sun light as much as possible and transform it to heat. For this purpose, extensive works have been performed by utilizing different kind of materials including metals,^7^, ^14^, ^15^, ^16^ carbon based materials,^17^, ^18^, ^19^, ^20^ organic polymers,^21^, ^22^ and semiconductor nanoparticles.^23^, ^24^, ^25^ Zhu et al. took use of “Black Au,” irregular Au particles which are black, realized an excellent solar vapor generation property, while the introducing of Au would increase the price. So the carbon materials especially the amorphous carbon materials are superb for their stability and cost compared with representative semiconductor nanoparticle, black titania, and metal nanoparticles.^24^, ^26^

Candle soot is an inexpensive carbon based amorphous hydrophobic nanomaterial with high surface area.^27^ When the soot is deposited on the substrates, hierarchical morphology can be obtained.^28^, ^29^, ^30^ Due to its hydrophobic nature, soot can endow substrates with superhydrophobic property. Meanwhile, light will be trapped within these hierarchical structures, thus decreasing the scattering and increasing the efficiency of light utilization.^31^, ^32^, ^33^

Inspired by these works, we deposited candle soot on one side of the cotton fabric to produce a self‐floating Janus solar absorber. Together with a polyester nonwoven protector, the as‐prepared Janus evaporator exhibited an efficiency of 86.3% under solar illumination of 1 kW m^−2^. In addition, due to the hydrophilic property of the cotton, salt can be quickly dissolved providing the material with salt‐rejecting property. Moreover, the as‐prepared evaporator performed well under low intensity and inclined radiation. More importantly, for preparing this equipment, no special apparatus as well as tedious process were needed. Finally, the cost of this Janus absorber is calculated as being less than $1 per m^2^. It is promising as portable solar vapor generator.

The process to prepare the Janus solar absorber is provided in the experimental section, and also simply described here. A cotton cloth was firstly wetted with water and then deposited with candle soot on one side of the fabric for different times. The soot particles analyzed by transmission electron microscope (TEM) are in the size of 30–50 nm as showed in **Figure** 1a. The D band appearing at 1350 cm^−1^ in Raman shift (Figure 1b) approves the amorphous structure for the soot. The amorphous soot layer can serve as a light absorber, and the interlaced fiber together with the close‐piled soot act as a hierarchical micro‐nanostructure to enhance the nonwettability. In addition, light scattering within such micro‐nanostructure is beneficial for enhancing light absorption. The sunlight absorption property can be confirmed by using an ultraviolet–visible–near infrared spectrophotometer in the range of 200–2500 nm (Figure 1c). The light absorptivity of the membrane can be calculated by using the equation: *A* = 1−*R*−*T* where *A* is the absorbance, *R* is the reflectance, and *T* is the transmittance. It can be seen that the absorbance of the membrane is higher than 95% in the visible range and above 90% in other regions. The other side of the fabric adsorbs water owing to the hydrophilic nature of the cotton. When heated, the water vapor can escape from the hydrophilic side to superhydrophobic side through the porous structure of the fabric (**Scheme** 1).

**Figure 1:**
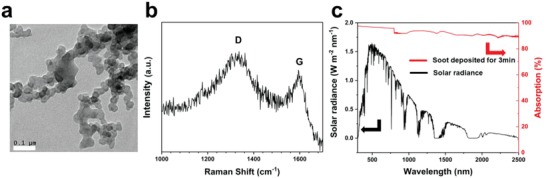
a) TEM images of the candle soot. b) Raman shift of the candle soot. The peaks at 1350 cm^−1^ (D bands) and the 1580 cm^−1^ (G bands) account for the amorphous carbon and the *E*_2g_ mode of graphite, respectively. c) The absorption spectra of the soot‐deposited fabric (deposition time of 3 min, red line) together with solar spectral irradiance (AM 1.5G, black line).

**Scheme 1:**
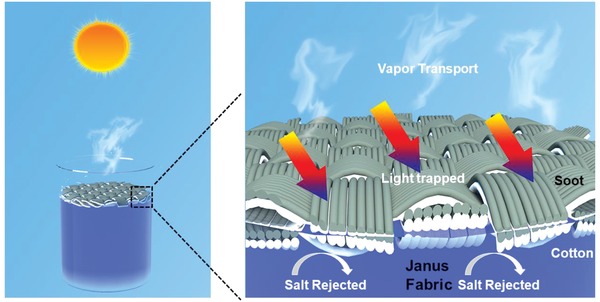
Schematic diagram of the soot‐deposited Janus fabric floating on the water surface.

We first investigated the Janus absorber without polyester cover. The influence of soot‐deposited time is shown in **Figure** 2a. It can be seen that the system with pristine cotton suspended in the water exhibited the worst ability under 1 sun radiation. This is because the white color cloth has reflected most of the light. In addition, the cotton cloth is fully immersed in the water, making the heating of water less efficient. With the deposition of the candle soot for 1 min, the mass change increased sharply and the evaporation rate was 2.2 times higher that of pure water. Further increase the deposition time to 3 min, the evaporation rate increased to 0.856 kg m^−2^ h^−1^, about 2.8 times that of pure water. We defined the thermal efficiency (η) as *η = m h*_LV_/*q_i_ C*_opt_, where *m* is the mass flux, *h*_LV_ is the liquid–vapor phase change enthalpy at 99 °C (2260 MJ kg^−1^), and *q_i_ C*_opt_ is the power density of solar irradiation.^34^, ^35^, ^36^, ^37^ The efficiency is calculated to be 53.7%. Compared with the similar Janus absorbs prepared by Zhu (≈51%) and Chen (57 ± 2.5%), soot‐deposited fabric demonstrated advanced performances, at the same time, demanding neither special equipment nor complex fabrication process.^38^, ^39^ It should be noticed that the natural evaporation rate is not excluded. That is because in the dark, the water gains heat from surroundings to realize spontaneous evaporation, while under solar illumination, the thermal energy flows inversely from the interface to the surroundings. So the evaporation rate obtained is used directly to evaluate the efficiency.^40^ At the deposition time of 5 min, the evaporation rate decreased slightly. This is due to the higher soot deposited on the surface, which blocked the vapor passage, a parameter that plays a critical role in the final performance.^41^

**Figure 2:**
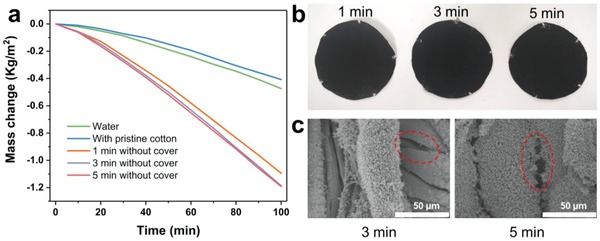
a) The mass change for absorber with different soot‐deposition time without the polyester protector under solar illumination of 1 kW m^−2^. b) The digital photograph of the absorber with different deposition time. c) The morphology of the absorber at different deposition time. The red dashed line pointed the interspace between fibers.

After incorporating a polyester cover, the mass change increased significantly (**Figure** 3a). At steady state, the vapor rate was 1.375 kW m^−2^ h^−1^ and the efficiency was 86.3% under solar illumination of 1 kW m^−2^. The function of the polyester is to decrease the heat loss of absorber that spread toward the surroundings.^42^, ^43^ The overall performance of our absorber is superior compared to other works (Table S1, Supporting Information). After soot deposited for 3 min, the front side of the flexible film resulted black (Figure 3b) with the soot and exhibited great superhydrophobic property having a contact angle (CA) of 159.7 ± 1.18° (Figure 3d). On the contrary, on the back side of the film, there was the natural white cotton (Figure 3c) with a CA of ≈0° (Figure 3d). On the superhydrophobic side, the mesoporous soot piled on the cotton fibers leading to a rough nanoscaled porous surface (Figure 3e). Meanwhile, the woven fibers provided microscaled voids (Figure 2c). When light reach the surface, it will scattering within such voids. So we believe that apart from the excellent light absorption of the carbon materials, the Janus structure can also contribute to such fast evaporate rate as well as high efficiency.

**Figure 3:**
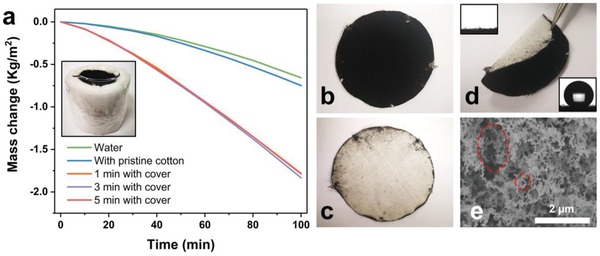
a) The mass change for absorber with different soot‐deposition time with the polyester protector under solar illumination of 1 kW m^−2^. b–d) The digital photograph of the b) front side, c) back side, and d) cross section of absorber with deposition time of 3 min. The insert were images of CA for the two sides. e) The morphology of the absorber with deposition time of 3 min. The red dashed line pointed the porous structure between soot.

In addition, the Janus structure guarantees the evaporator to float on the water that can focus the heat on the surface. As shown in **Figure** 4a, at the beginning of the experiment, the temperature of water is 21.3 °C which is the same as the surrounding. And there is no difference in vertical direction for the water. When being heated, the temperature of the water increased with a gradient observed along the vertical direction. This confirms that the light was focused on the top layer with soot during exposure. Moreover, the superhydrophilic side is superaerophobic under water, the vapors tend to leave the downside of the fabric to upside which can also increase the evaporation rate.^44^ At the end of the experiment, the temperature of the interface reaches ≈40 °C. Convention heat loss ratio (*R*_c_) and radiation heat loss ratio (*R*_r_) are two relevant parameters to be measured for estimating the efficiency of the system. According to the classical theory, we define *R_c_* = *α ΔT S*/*q_i_ C*_opt_ where α is the convective heat transfer coefficient for air, *ΔT* is the temperature difference between the surface and surroundings and *q_i_ C*_opt_ is the power density of solar irradiation. Whereas the radiation heat loss ratio is defined as *R_r_ = C_n_* (*T*_1_^^^4−*T*_2_^^^4)/ *q_i_ C*_opt_, *C_n_* is radiation coefficient of air, *T*_1_ and *T*_2_ are the temperature of the surface and the surroundings, respectively, *q_i_ C*_opt_ also represents the power density of solar irradiation. We found that in our system the convention heat loss is around 11% and the radiation heat loss is about 14%. Considering that our efficiency is 86% (82.11 ± 4.38%, average efficiency for 5 cycles, Figure S1a, Supporting Information) and the total efficiency is more than 100%, it seems that our result breaks the law of conservation of energy. To explain this phenomenon, we measured the temperature of a piece of paper settled 1 cm above the surface. As shown in Figure S1b,c in the Supporting Information, there is a stable and even layer having a temperature of 31 °C, which is different from the same position above a hot stage. Considering that the hot stage and the surface of absorber share the same temperature, the only difference between them is that apart from air, there are lots of vapors as well as waters that liquefied from vapors above the surface of the absorber. Thus a reasonable explanation can be draw. When vapors rise up, it quickly cool down and liquefy. At the same time, it releases a large amount of heat which endows such “vapor–water–air” hybrid layer with a compared high temperature. Besides, due to the great specific heat capacity of the water, this hot layer can be stable and thus acts as a perfect cushion, decreasing the heat loss of the absorber and increasing the efficiency.

**Figure 4:**
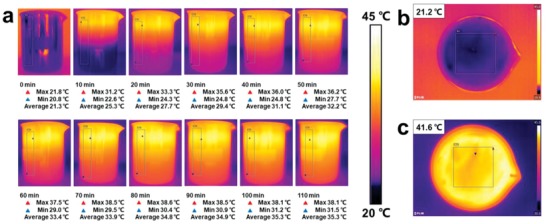
a) The IR thermal images of water with different light irradiation time from a side direction under solar illumination of 1 kW m^−2^. b,c) The IR thermal images of absorber b) before and c) after irradiation.

The strength and the incident angle of the solar light always change during the day, so it is of great importance to test the efficiency under lower solar fluxes, as well as mass change under different incident angle. It could be seen in **Figure** 5a, due to the lower evaporation temperatures, the sufficiency under low solar strength is lower than that under 1 kW m^−2^, while it is still larger than 70% under 0.7 sun.^39^ That means the equipment can be used in the cloudy days.

**Figure 5:**
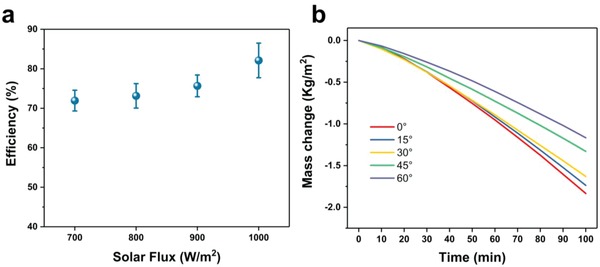
a) The efficiency under different solar flux. The error bar for the strength of 700–900 W m^−2^ was calculated tree times, and 5 times for strength of 1 kW m^−2^. b) The mass change with different incident angle under solar illumination of 1 kW m^−2^.

Figure 5b shows that the mass change decreased with the increase of the incident angle, while a relatively small change when the angle is within 20–30°. The isotropic distribution of soot can harvest light efficiently for incident light from all directions, thus lead to an eminent performance.^41^, ^45^ To test the stability of the absorber, a piece of soot‐deposited cotton was placed in water and then treated with an ultrasonic machine (40 kHz). As shown in Video S1 in the Supporting Information, the Janus structure endows the fabric with a prominent floating stability even if it was under strong ultrasonic treatment for 5 min. Besides, there was almost no soot shed off during the test, even though there is only physic bond between the soot and the fabric. So we can confirm that the Janus structure endow the absorber with both a great self‐floating ability and an ability to withstand such a harsh environment. That means the absorber will be an outstanding candidate for outdoor usage. (Figure S2, Supporting Information).

Due to the hydrophilic property of the cotton, the as‐prepared absorber also possessed excellent salt rejection capability. Some experiments were carried to confirm it. Firstly, the mass change of the pure water as well as simulated seawater (3.5 wt% NaCl) was recorded under the same condition (18 °C, 17% relative humidity, 1 sun). The result shows no difference between them (**Figure** 6a). Moreover, long time evaporation experiment was also performed in simulated seawater to test the stability.^39^, ^46^, ^47^ The equipment was exposed in a 3.5 wt% NaCl solution for 10 days. In each day, it was exposed to 5 h of solar irradiation at strength of 1 kW m^−2^, the system was allowed to cool down and reject salt in the remaining time. Figure 6b shows the mass change of the salt water. Considering that the room temperature and humidity is slightly different every day, we infer that the difference in mass change during the 10 days was not relevant. Besides, a continuous irradiation test was also carried under 1 sun with 3.5 wt% NaCl solution as simulated seawater. There is no obvious NaCl crystal appeared on the surface of the absorber after 100 h (Figure S3, Supporting Information). Therefore, we confirm the good salt rejection capability for the equipment.

**Figure 6:**
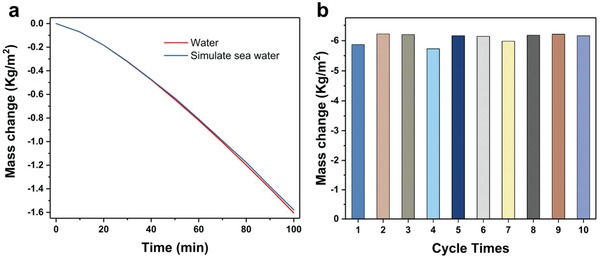
a) The mass change of the pure water is similar with that for simulated seawater (3.5 wt% NaCl) under the same condition (18 °C, 17% RH). b) The mass change for 10 day cycles under solar illumination of 1 kW m^−2^.

The seawater from Yellow Sea was employed to further assess the desalination ability. As shown in **Figure** 7, the seawater concentration of four ions (Na^+^, K^+^, Ca^2+^, and Mg^2+^) decreased sharply at a level far below the safe‐drinking‐water‐values given by World Health Organization (WHO) where concentration of Na^+^ is required less than 200 mg L^−1^ and the sum of Ca^2+^, Mg^2+^ is less than 500 mg L^−1^.^36^

**Figure 7:**
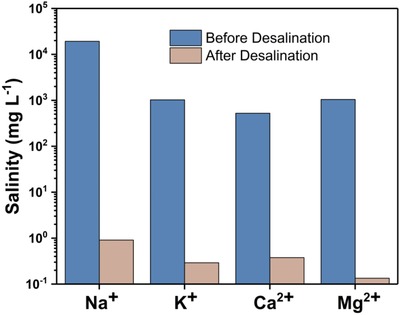
The concentrations of four kind of primary ions in seawater (Na^+^, K^+^, Ca^2+^, and Mg^2+^) before and after desalination.

Furthermore, the cost of the evaporation structure was estimated. The plain cotton with mass per unit of 105 g m^−2^ is about $ 0.55, together with candle the total cost is less than $1 per m^2^ (the price is from local market and the exchange rate is based on the exchange rate on May 23, 2018). Such low cost clearly favors practical applications.

In conclusion, inspired by lotus leaf, we fabricated self‐floating Janus solar absorber from cotton cloth for sustainable clean water collection through vaporization. The soot was selectively deposited on one side of the cotton fabric, while the other side remained uncoated. The hierarchical porous structure on the coated side and the contrasting wetting properties of the two sides has enabled a good efficient for water vapor transport through the Janus cloth under solar illumination. This simple setup worked well with salt water, which provides a new way for efficient water desalination. With no special apparatus needed, no tedious process required, and a cost less than $1 per m^2^, this flexible Janus absorber is promising as portable solar vapor generator.

## Experimental Section

*Materials*: The cotton fabric was purchased from Shanghai Textile Industry. The candle and the polyester nonwoven cover (thick of 0.5 cm, folded 3 times) were bought from a local market in Suzhou. The NaCl was obtained from Chinasun Specialty Products Co., Ltd.

*Preparation of Janus Substrates*: The circular cotton fabric with diameter of 5 cm was wetted with water. It was then used to capture candle soot when exposed to candle flame for different times.

*Characterization*: The morphology was analyzed by a field emission scanning electron microscope (FESEM, Hitachi S‐4800). KRÜSS DSA 100 (KRÜSS, Germany) was employed to test the CA. Each sample was tested 5 times. It was used 6 µL of water. The absorption spectra were measured by an ultraviolet–visible–near infrared spectrophotometer (Agilent, Cary 5000). Raman shift spectra were obtained using a Raman spectrometer (HORIBA, JOBIN YVON, FM4P‐TCSPC). The TEM imagines were taken by using a TEM (FEI Tecnai G‐20 operated at 200 kV). The salinity was obtained by an inductively coupled plasma optical emission spectrometry (Varian, vista‐MPX). Hot stage (Cossim, KER3100‐08S) was employed to heat the paper 1 cm above the surface.

*Photothermal Evaporation Experiments*: The absorber was placed under a sunlight simulated illumination source (CEL‐HXF300, CEAULIGHT with AM 1.5 optical filter). And the mass changed was recorded by electronic scales (Ohaus, Scout Pro). The illumination strength was measured by an accessional detector (CEL‐NP2000, CEAULIGHT). The infrared radiation pictures and surface temperature were recorded by using an IR camera (FLIR T620).

## Conflict of Interest

The authors declare no conflict of interest.

## Supporting information

Supplementary
