# Supplementary material for: Bioinspired Soot‐Deposited Janus Fabrics for Sustainable Solar Steam Generation with Salt‐Rejection
Source: Glob Chall. 2019 Feb 10;3(8):1800117. doi: 10.1002/gch2.201800117 (PMC6686278; doi:10.1002/gch2.201800117)
Supplement: Supplementary file 1 — Supplementary [file GCH2-3-1800117-s001.pdf]

Copyright WILEY-VCH Verlag GmbH & Co. KGaA, 69469 Weinheim, Germany, 2019.

Global Challenges
Open Access

Supporting Information

for *Global Challenges,* DOI: 10.1002/gch2.201800117

Bioinspired Soot-Deposited Janus Fabrics for Sustainable Solar Steam Generation with Salt-Rejection

*Shouwei Gao, Xiuli Dong, Jianying Huang, Jianing Dong, Francesco Di Maggio, Shanchi Wang, Fang Guo, Tianxue Zhu, Zhong Chen, and Yuekun Lai**

Copyright WILEY-VCH Verlag GmbH & Co. KGaA, 69469 Weinheim, Germany, 2016.

Supporting Information

Bioinspired soot-deposited Janus fabrics for sustainable solar steam generation with salt-rejection

*Shouwei Gao, Xiuli Dong, Jianying Huang, Jianing Dong, Francesco Di Maggio, Shanchi Wang, Fang Guo, Tianxue Zhu, Zhong Chen, and Yuekun Lai**

S. W. Gao, X. L. Dong, J. N. Dong, S. C. Wang, F. Guo, T. X. Zhu, Prof. Y. K. Lai
National Engineering Laboratory for Modern Silk, College of Textile and Clothing Engineering, Soochow University, Suzhou 215123, P. R. China
E-mail: yklai@suda.edu.cn
Prof. J. Y. Huang, Prof. Y. K. Lai
College of Chemical Engineering, Fuzhou University, Fuzhou 350116, P. R. China
Prof. Z. Chen
School of Materials Science and Engineering, Nanyang Technological University, 50 Nanyang Avenue, Singapore 639798, Singapore
Dr. F. D. Maggio
Division of Medicine, University College London, London WC1E 6JF, UK

Figure captions:

Figure S1. (a) The mass change for the vapor process for 5 cycles. (b-c) The IR thermal images of a piece of paper placed 1 cm above the surface of surface of absorber (b) and hot stage (c).

Figure S2. The images of the Janus fabric before (a) and after (b) ultrasonic treatment for 5 min.

Figure S3. The images and the corresponding SEM imagines for the surface of Janus fabric before (a) and after continuous irradiation under 1 sun for 100 h with 3.5 wt% NaCl solution as simulated sea water (b).

Table S1. The overall performance of pervious works.

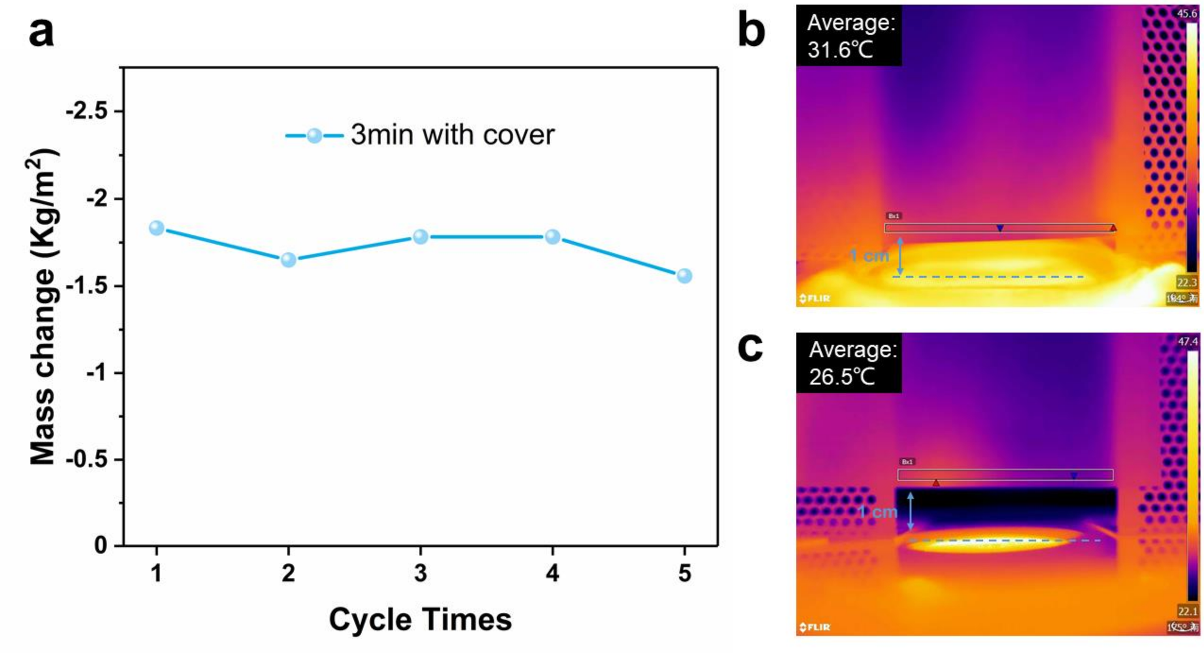

Figure S1. (a) The mass change for the vapor process for 5 cycles. (b-c) The IR thermal images of a piece of paper placed 1 cm above the surface of surface of absorber (b) and hot stage (c).

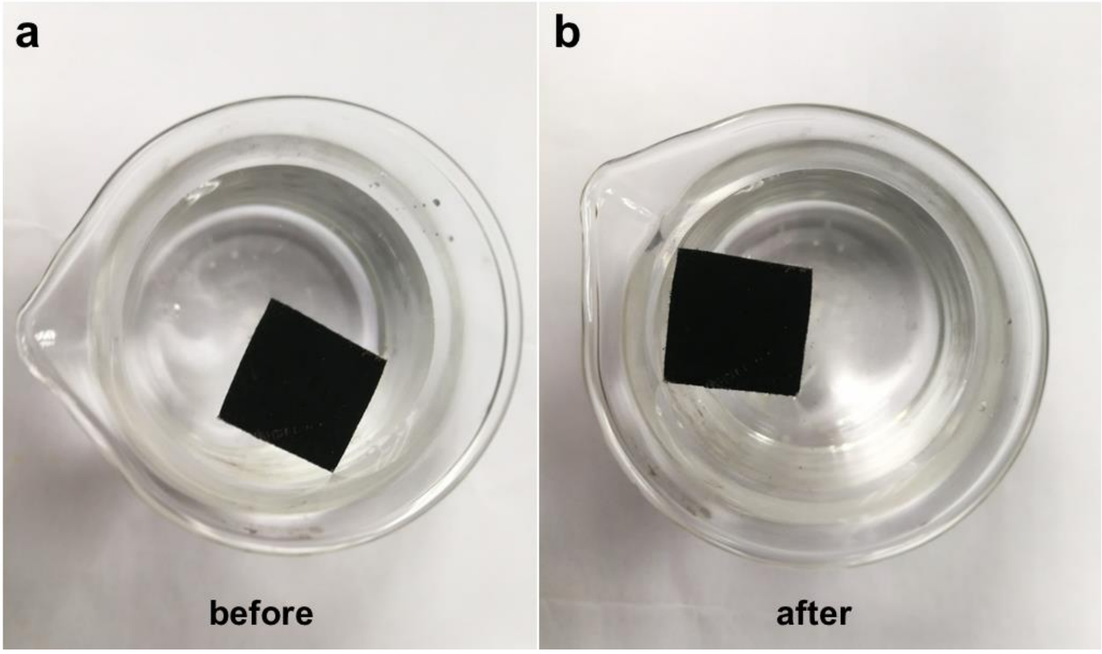

Figure S2. The images of the Janus fabric before (a) and after (b) ultrasonic treatment for 5 min.

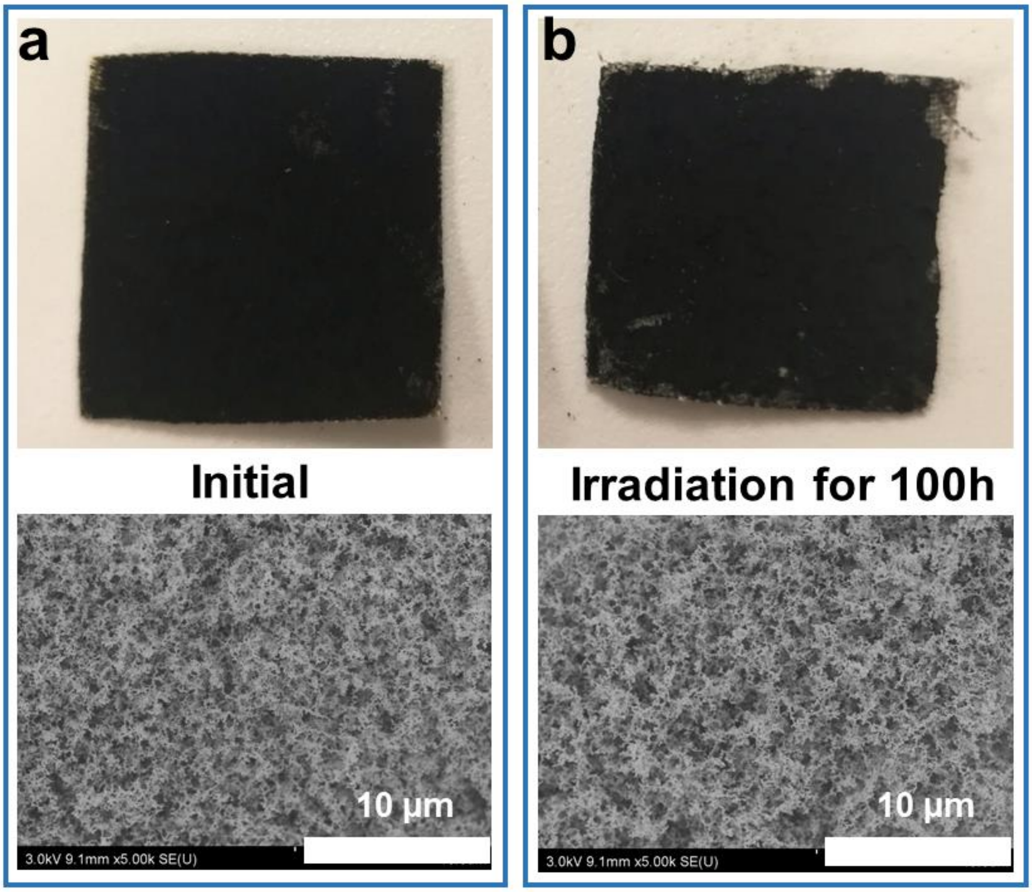

Figure S3. The images and the corresponding SEM imagines for the surface of Janus fabric before (a) and after continuous irradiation under 1 sun for 100 h with 3.5 wt% NaCl solution as simulated sea water (b).

Table S1. The overall performance of pervious works.

**Device**	Mass change (kg m^{-2} h^{-1})	Solar intensity (kW m^{-2})	Energy conversion efficiency (%)	Ref.
Black TiO_x/stainless steel mesh	0.801	1	50.3	1
Al NP/AAM	2	2	70	2
Mushrooms	1.475	1	78	3
CB/PMMA & PAN Janus absorber with insulation	1.3	1	72 (51 without insulation)	4

Carbonized melamine	1.270	1	87.3	5
rGO/PU foam	0.9	1	65	6
Nanotube-modified flexible wood	0.95	1	65	7
Au NP/AAM	/	4	65	8
Black fabric Janus absorber with insulation	/	1	57	9
**Soot/cotton Janus absorber with cover**	**1.375 (0.856 without cover)**	**1**	**86.3 (53.7 without cover)**	**This Work**

Reference

[1] M. Ye, J. Jia, Z. Wu, C. Qian, R. Chen, P. G. O'Brien, W. Sun, Y. Dong, G. A. Ozin, *Adv. Energy Mater.* **2017**, *7*, 1601811.

[2] L. Zhou, Y. Tan, J. Wang, W. Xu, Y. Yuan, W. Cai, S. Zhu, J. Zhu, *Nat. Photonics*. **2016**, *10*, 393.

[3] N. Xu, X. Hu, W. Xu, X. Li, L. Zhou, S. Zhu, J. Zhu, *Adv. Mater.* **2017**, *29*, 1606762.

[4] X. Lin, J. Chen, Z. Yuan, M. Yang, G. Chen, D. Yu, M. Zhang, W. Hong, X. Chen, *J. Mater. Chem. A* **2018**, *6*, 4642.

[5] X. Lin, J. Chen, Z. Yuan, M. Yang, G. Chen, D. Yu, M. Zhang, W. Hong, X. Chen, *J. Mater. Chem. A* **2017,** *6*, 4642-4648.

[6] G. Wang, Y. Fu, A. Guo, T. Mei, J. Wang, J. Li, X. Wang, *Chem. Mater.* **2017**, *29*, 5629.

[7] C. Chen, Y. Li, J. Song, Z. Yang, Y. Kuang, E. Hitz, C. Jia, A. Gong, F. Jiang, J. Y. Zhu, B. Yang, J. Xie, L. Hu, *Adv. Mater.* **2017**, *29*, 1701756.

[8] L. Zhou, S. Zhuang, C. He, Y. Tan, Z. Wang, J. Zhu, *Nano Energy* **2017**, *32*, 195.

[9] G. W. Ni, S. H. Zandavi, S. M. Javid, S. V. Boriskina, T. A. Cooper, G. Chen, *Energy Environ. Sci.* **2018,** *11*, 1510-1519.
